# Exploring heterogeneous profiles of academic performance among college students: a latent profile and LASSO regression analysis

**DOI:** 10.3389/fpsyg.2026.1732350

**Published:** 2026-05-13

**Authors:** Hui Li, Rongrong Guan, Hongyun Zhao, Huiyang Han, Xianfeng Li, Zhenti Cui

**Affiliations:** 1 School of Medicine, Sias University, Zhengzhou, China; 2 School of Art and Design, Zhengzhou University of Industrial Technology, Zhengzhou, China; 3 School of Engineering, Sias University, Zhengzhou, China

**Keywords:** academic performance, college students, influencing factors, LASSO, LPA

## Abstract

**Objective:**

With the development of higher education, college students face various psychological pressures and challenges. Consequently, academic performance has become a key focus of educational research. This study aims to identify latent categories of academic performance among college students and further explore risk factors associated with these categories.

**Methods:**

Employing a convenience sampling strategy, 548 undergraduate students were recruited from universities in Henan Province between May and July 2025, the effective response rate is 94%. Data collection utilized the Family Affluence Scale-III, General Self-Efficacy Scale, Perceived Social Support Scale, Academic Performance Scale, General Procrastination Scale, Utrecht Work Engagement Scale-Student, and demographic questionnaire. Three academic performance-related scales served as observed variables. Latent profile analysis (LPA) identified latent categories of academic performance among college students. Univariate analyses using Chi-square tests, Mann–Whitney U tests, or Fisher’s exact tests compared characteristics and differences across academic performance categories. Collected variables were incorporated into a relaxed least absolute shrinkage and selection operator (LASSO) method based on bootstrap resampling to confirm key factors influencing these categories. Logistic regression analysis was then conducted on the identified factors.

**Results:**

Two latent categories of academic performance among college students were identified: “Low Self-Regulators” and “High Self-Regulators.” After variable selection via Bootstrap Relaxed LASSO regression, with the “High Self-Regulators” serving as the reference group, logistic regression analysis revealed that general self-efficacy (OR = 0.79, 95% CI = [0.73–0.85], *p* < 0.001), perceived social support (OR = 0.93, 95% CI = [0.87–0.98], *p* < 0.001), and purposeful exercise frequency (OR = 0.81, 95% CI = [0.64–1.00], *p* < 0.001) were significant factors influencing the latent categories of academic performance among college students.

**Conclusion:**

This study adopted a person-centered approach and identified two latent categories of college students’ academic performance through latent profile analysis: “Low Self-regulators” (average academic performance and engagement, higher procrastination) and “High Self-regulators” (higher academic performance and engagement, lower procrastination). Further screening via Bootstrap relaxed LASSO regression revealed that general self-efficacy, perceived social support, and purposeful exercise frequency were the most stable predictors of student category membership. These findings provide empirical support for implementing differentiated academic interventions in higher education, suggesting educators should precisely enhance students’ academic adaptation and effectiveness by addressing the psychological needs of distinct groups through boosting self-efficacy and strengthening social support systems.

## Introduction

Amidst the current global expansion of higher education and intensifying competition (Mok et al., 2022; Frank and Meyer, 2024; Tomlinson and Watermeyer, 2022), college students face increasing academic challenges and pressures (Tomlinson and Watermeyer, 2022; Barbayannis et al., 2022). Consequently, their academic performance has become a critical focus for educational research and university administration. Under the weight of course loads, competitive environments, and uncertainties surrounding future career development, academic performance is not merely a matter of grades but is also closely linked to psychological factors, motivation, and self-regulation (Howard et al., 2021). Moreover, suboptimal academic performance not only undermines academic confidence and willingness to pursue further studies but may also lead to reduced self-efficacy, anxiety, depression, and other mental health issues (Agnafors et al., 2021). Therefore, identifying distinct groups of college students based on their academic performance types and delving into the underlying psychological mechanisms is crucial for both educational practice and policy development.

To the underlying psychological mechanisms of these distinct academic groups, this study is grounded in Self-Regulated Learning (SRL) theory (Zimmerman, 2000), which emphasizes that academic success stems in a complex interplay from individual cognitive, motivational, and behavioral strategies. Within this theoretical framework, self-efficacy is identified as a core motivational component, originally proposed by Bandura in 1977, self-efficacy refers to an individual’s dynamic belief in their ability to accomplish specific tasks, the research indicates that self-efficacy significantly influences college students’ learning engagement, learning strategies, and academic performance (Bandura, 1977). For instance, Zhao’s research found that college students with high academic self-efficacy and positive coping strategies exhibited significantly higher academic satisfaction and learning engagement compared to other groups (Zhao, 2024). Concurrently, other studies have confirmed the close relationship between self-efficacy and learning motivation, emotional regulation, and mental health (Schunk and DiBenedetto, 2020). Collectively, these studies demonstrate that self-efficacy serves as a crucial psychological resource for academic success, making it a representative example among the key influencing factors identified in this research.

However, most existing studies employ variable-centered approaches (Wu et al., 2021). While revealing overall trends, this methodology often overlooks potential heterogeneity within student populations (Howard and Hoffman, 2018). In fact, different students may exhibit distinct patterns of combination between self-efficacy and academic performance. For instance, some students exhibit high self-efficacy but insufficient learning engagement, while others exhibit low academic procrastination alongside low self-efficacy. These group differences are often overlooked in traditional analyses.

In recent years, the field of educational psychology has shown increasing interest in latent profile analysis (LPA) methods. Through LPA, researchers can uncover latent categories of students based on psychological variables, motivation, and learning behaviors, thereby gaining a more comprehensive understanding of group differences. For instance, Zhao used LPA to categorize college students into four groups based on academic stress, self-efficacy, and coping strategies (low-spirited, general copers, capable but passive, and optimistic and confident), revealing significant differences in academic satisfaction and engagement across these categories (Zhao, 2024). Other scholars have identified multiple groups of college students based on self-management and self-monitoring, revealing significant differences in motivation, mental health, and academic outcomes across these groups (Zhao and Ling, 2022). These studies demonstrate that LPA provides an effective tool for revealing group heterogeneity. Therefore, introducing LPA, an individual-centered approach aids in identifying distinct latent categories of academic performance among college students, thereby facilitating a more comprehensive understanding of the underlying psychological mechanisms.

Still, current research in this direction has limitations. First, while existing research has addressed self-efficacy, motivation, and strategy use, at the level of analysis it has tended to be limited to a single scale for categorization, with profiles constructed on the basis of self-efficacy scales (Wang et al., 2021), and has lacked a multidimensional, systematic framework across constructs. Second, few studies use scores from multiple academic-related scales as composite indicators to delineate latent categories. Third, while existing research has revealed significant correlations between self-efficacy and academic performance, systematic identification and validation are still needed to determine which core factors can effectively distinguish different academic performance types, particularly the key risk factors that lead students into academic difficulties.

Recent studies offer insights into filling these gaps. For instance, Zhou et al. employed LPA to examine learning engagement and anxiety levels among high school students, revealing that anxiety and engagement can form multiple combinatorial categories, with distinct categories showing significant differences in academic adjustment (Zhou et al., 2025). This suggests that using multi-scale indicators among college students can similarly uncover heterogeneous categories of academic performance. On the other hand, Vos et al. applied LPA to a sample of distance learners to examine how self-regulated learning, self-regulatory efficacy, motivation, and psychological need fulfillment influence learning behavior categories, finding significant differences in learning outcomes with different categories (Vos et al., 2025). These studies demonstrate that integrating multiple psychological scales into LPA is an effective approach to understanding students’ differentiated performance.

Given that traditional “variable-centered” research struggles to capture the heterogeneity of this interplay, employing “person-centered” LPA to identify distinct academic regulation subgroups has become a significant trend (Hertel et al., 2024). Therefore, this study first employs LPA, which is based on scales measuring academic performance, procrastination, and engagement, to categorize college students into latent groups. To address potential multicollinearity from numerous influencing factors and robust feature selection, this study innovatively employs bootstrap relaxed least absolute shrinkage and selection operator (LASSO) regression for key variable screening, rather than a standard regression analysis. This approach allows us to assess the stability of risk factors and derive robust odds ratios with bootstrap confidence intervals. Ultimately, this method precisely identifies critical risk factors determining students’ assignment to different academic performance categories, providing empirical evidence for targeted interventions and personalized tutoring in higher education.

## Theoretical background

### Self-regulated learning and self-efficacy

This study is based on the Self-Regulated Learning (SRL) theory, which views academic success as the result of a dynamic interplay between cognitive, motivational, and behavioral strategies (Zimmerman, 2000). Within this theoretical framework, self-efficacy plays a role as a motivational mechanism (Bandura, 1997). Bandura defines self-efficacy as an individual’s belief that he or she has the ability to organize and carry out the actions required to achieve a goal. As a general rule, people with high self-efficacy will view difficult tasks as challenges to be overcome rather than threats to be avoided (Bandura, 1994). In the field of higher education, such beliefs have an important precedent. Students with high self-efficacy are more likely to sustain effort, manage their time effectively, and recover quickly from setbacks, whereas lower self-efficacy tends to lead to maladaptive behaviors such as anxiety and avoidance (Chemers et al., 2001).

### Multidimensional regulatory system

SRL is not a single trait but is manifested through different moderating dimensions. In this study, we considered three key dimensions that reflect self-regulatory outcomes. Firstly, learning engagement (motivational state) is characterized by vigor, concentration, and full attention and represents the positive psychology invested in the learning process (Schaufeli et al., 2002). In addition, academic procrastination (volitional failure) is the result of a breakdown in self-regulation that leads to the voluntary postponement of intended actions, which directly hinders engagement (Steel, 2007). Finally, Adaptive learning behavior (strategy execution) is measured by the Academic performance scale (APS). This dimension focuses on the specific implementation of the strategy, such as active preparation, timely initiation, rather than purely on the outcome grade.

### The person-centered approach

#### Paradigm shift: from variables to individuals

Although variable-centered approaches can describe average relationships among factors, they often overlook the heterogeneity within student populations. Person-centered approaches shift the analytical focus to individuals, addressing this limitation. This methodology posits that psychological attributes do not operate in isolation but form unique configurations within each individual (Spurk et al., 2020). From a holistic perspective, the person-centered approach enables researchers to transcend average effects, identifying how varying levels of self-efficacy and self-regulation combine to manifest as distinct, heterogeneous learner profiles (Nie et al., 2024).

#### Theoretical integration of SRL profiles

Adopting an individual-centered approach is crucial for capturing the heterogeneity of motivational states and behavioral outcomes. For instance, learners with similar background characteristics may exhibit markedly different patterns of academic functioning when observed through a profile-based perspective (Söderholm et al., 2023). This approach enables the identification of vulnerable subgroups, such as students who may possess specific motivational strengths but are hindered by regulatory deficits, facilitating the development of more tailored pedagogical interventions (Nie et al., 2024), as similarly demonstrated in research on student engagement profiles (Söderholm et al., 2023).

## Methods

### Sample size

This cross-sectional study was conducted among college students in Henan Province from May to July 2025. Participants were recruited from universities in Henan Province using convenience sampling. Approximately 90% of the samples came from students at a comprehensive university in Henan Province, while the remaining participants came from other higher education institutions in the area. The questionnaire was sent out online using a tool called Microsoft Forms. It was mostly shared through WeChat and SuperStarLearn. Simultaneously, the sample size was determined based on the minimum requirements for LPA (N ≥ 500; Nylund-Gibson et al., 2023). The inclusion criteria were: (Mok et al., 2022) undergraduate students enrolled in Henan Province universities; (Frank and Meyer, 2024) voluntary participation in this study with informed consent. During data cleaning, we corrected an outlier in the political beliefs responses and merged the two fifth-year undergraduate samples, combining them with the fourth-year undergraduates as senior students. Additionally, we excluded 10 questionnaires completed in under 1 min and 30 s, as well as 21 questionnaires completed in under 120 s that exhibited a straight-line response pattern across the five core scales. We collected 548 questionnaires and ultimately used 517 responses, the effective response rate is 94%.

### Instruments

#### General demographic information collection table

A self-designed data collection form, including gender, ethnicity, political belief, academic year, high school track, number of Gaokao attempts, scholarships (past year), English proficiency, student leadership experience, current student leadership position, majors change, military service, volunteer service, late bedtime frequency, purposeful exercise frequency, father’s educational attainment, and mother’s educational attainment. These variables were selected to comprehensively capture factors previously identified in research as influencing academic performance, including socioeconomic status (Zhang et al., 2020), lifestyle habits (Hysing et al., 2016; Barbosa et al., 2020), and student leadership experiences (Aquino et al., 2025).

#### Latent profile indicators

The Academic Performance Scale (APS; Cronbach’s *α* = 0.90) was developed by McGregory (2015) and translated into Chinese by the team of this study. The General Procrastination Scale (GPS; Cronbach’s *α* = 0.92) was developed by Lay (1986), with its Chinese version revised by Cuiqiu (2007). Considering the factor loadings from the confirmatory factor analysis, this study excluded nine items with excessively low or negative loadings, retaining 11 positively scored items.

The Utrecht Work Engagement Scale-Student (UWES-S; Cronbach’s *α* = 0.94) was developed by Schaufeli et al. based on the Utrecht Work Engagement Scale (UWES) (UWES, 2003), with its Chinese version revised by Li and Huang (2010).

#### Predictor scales

The Family Affluence Scale-III (FAS-III; Cronbach’s α = 0.67) was developed by the World Health Organization’s Health Behaviors in School-aged Children team. Although originally designed for adolescents, FAS-III remains applicable to college students who remain financially dependent on their families, as family affluence influences their behavioral pathways similarly.

The General Self-Efficacy Scale (GSES; Cronbach’s α = 0.93) was developed by professor Ralf Schwarzer and his team of clinical and health psychologists in Germany (Schwarzer et al., 1995), with the Chinese version revised by Zhang and Schwarzer (1995).

The Perceived Social Support Scale (PSSS; Cronbach’s α = 0.92) was developed by Zimet et al. (1990). The Chinese version was translated by Jiang (2001). This study employed PSSS revised by Yan based on Jiang’s translation (Yan et al., 2011).

In this study, the FAS-III Cronbach’s alpha coefficient was 0.67, potentially due to the scale’s relatively few items. Boer et al. noted in their research that Cronbach’s alpha coefficients for FAS items in most countries range between 0.60 and 0.70, yet these values remain acceptable indicators of internal consistency (Boer et al., 2024). Except for FAS-III, which was used for the first time with college students and thus required exploratory use, all other scales already had existing normative data for Chinese college students.

The study employed six self-report scales with good reliability and validity. Except for the FAS-III, which used cumulative scoring, all scales were scored using a Likert scale. All scale items were confirmed to be unambiguous in a pre-survey. Detailed details are presented in Table 1.

**Table 1:** summary of measurement instruments.

Instrument name	Title item	Scoring	Score range	Cronbach’s alpha (in this study)	Structure	Example item
FAS-III	6	0/1/2/3	0–13	0.67	C	Does your family have a dishwasher?
GSES	10	1–4	10–40	0.93	U	I can always manage to solve difficult problems if I try hard enough.
PSSS	12	1–7	12–84	0.92	Family support/Friend support/other support	My family really tries to help me/ I can count on my friends when things go wrong/ There is a special person who is around when I am in need
APS	8	1–5	8–40	0.90	U	I pay attention and listen during every discussion.
GPS	11	1–5	11–55	0.92	U	I often find myself performing tasks that I had intended to do days before.
UWES-S	17	1–7	17–119	0.94	Vigor/Dedication/Absorption	At my class, I feel bursting with energy/ I find my studies full of meaning and purpose/ Time flies when I am studying.

FAS-III, indicators of material wealth, higher scores are higher family affluence. GSES, high scores are high self-efficacy. PSSS, high scores are strong social support. APS, first Chinese localization, higher scores are better performance. GPS, high scores are high procrastination. UWES-S, high scores are high academic engagement. C, this scale serves as a composite indicator. U, this is a unidimensional scale.

### Ethics statement

The studies involving humans were approved by the Ethics Committee of School of Medicine, Sias University (project number: LL-2025-QLJH-09). The studies were conducted in accordance with the local legislation and institutional requirement and with the Declaration of Helsinki. Online informed consent for participation in this study was provided by the participants.

### Statistical analyses

#### Descriptive statistics and measurement assessment

Descriptive statistics summarize the general characteristics of participants. Categorical variables are presented as counts and percentages, while continuous variables not following a normal distribution are represented by median and interquartile range. Univariate analyses were conducted using Chi-square tests, Mann–Whitney U tests, or Fisher’s exact tests to compare characteristics and differences across academic performance categories. Statistical analyses were conducted using R software (version 4.2.3), with a two-tailed significance threshold of *p* < 0.05.

To examine the construct validity of each measurement tool, we conducted confirmatory factor analysis (CFA). Parameter estimation employed robust maximum likelihood (MLR) estimation. Model fit criteria were established as follows: *χ^2^/df*<3 or 5, comparative fit index (CFI) and Tucker-Lewis index (TLI) > 0.90, root mean square error of approximation (RMSEA) and standardized residual mean square (SRMR) < 0.08 (Hu and Bentler, 1999). The results indicated a satisfactory model fit for the measurement model (see Supplementary Table S1).

Additionally, we calculated composite reliability (CR) and average variance extracted (AVE; Fornell and Larcker, 1981). Values of CR > 0.70 and AVE > 0.50 indicate good internal consistency and convergent validity for the measurement tools. We also computed Cronbach’s alpha coefficients for each variable as supplementary reliability indicators. The analysis confirmed that all the tools used for measurement met these psychometric standards (see Supplementary Table S2). Spearman correlation analysis revealed complex relationships among variables (see Supplementary Table S3). The relationship between self-efficacy and general procrastination was not significant (*r* = 0.03, *p* > 0.05) at the aggregate level. This lack of a simple linear association hints at unobserved heterogeneity within the population, thereby supporting the use of a person-centered approach to identify distinct subgroups.

Finally, as the study data relied on self-reports, common method bias (CMB) was a potential concern. Therefore, a single-factor test was employed for statistical control (Podsakoff et al., 2003). This involved constructing a restrictive model loading all measurement items onto a single latent factor and comparing its fit indices against the hypothesized multi-factor measurement model. The results showed that the single-factor model was not a good fit and was much worse than the multi-factor model. This suggests that there was not a lot of common method bias (see Supplementary Table S1). These measurement assessments are primarily implemented using the R packages lavaan and semTools.

#### Latent profile analysis

LPA identifies latent categories of academic performance among college students using standardized scores from the APS, GPS, and UWES-S. The analysis begins with the assumption of one latent class, progressively increasing the number of classes until model fit metrics cease to improve significantly. To minimize the risk of overparameterization and ensure stable model convergence, we employ a class-invariant, diagonal parameterization scheme for latent profile analysis, commonly referred to as Model 1 (Pastor et al., 2007). In this Model 1, the variance of indicators is constrained to be equal across profiles, and the covariance between indicators is fixed to zero. This setting is predicated on the assumption of local independence, with the hypothesis being put forward that the dispersion of indicators is consistent across different subgroups.

In determining the optimal number of latent classes, we primarily referenced the following fit indices: Akaike Information Criterion (AIC), Bayesian Information Criterion (BIC), and adjusted Bayesian Information Criterion (aBIC). By balancing model complexity and goodness-of-fit, lower values of these metrics indicate better model fit (Fonseca-Pedrero et al., 2017; Li et al., 2020; Marsh et al., 2004). Additionally, entropy is used to measure classification accuracy, ranging from 0 to 1, where values closer to 1 indicate greater precision. An entropy value exceeding 0.8 is crucial for the reliability of interpretable models, simultaneously, the Lo–Mendell–Rubin Adjusted Likelihood Ratio Test (LMRT) and Bootstrapped Likelihood Ratio Test (BLRT) were employed to compare the fitting differences among models with varying latent categories. A *p*-value below 0.05 indicates that the k-category model significantly outperforms the k − 1 category model (Tein et al., 2013). This analysis was conducted in the R environment using packages such as tidyLPA.

#### Predictors of latent profile membership analysis

To identify robust predictor variables and obtain unbiased effect estimates, this study employed a relaxed LASSO method based on bootstrap resampling. The process comprised 1,000 bootstrap iterations, each executing two stages. During the variable selection phase, logistic LASSO regressions were run on bootstrap samples. Cross-validation determined the optimal penalty parameter, filtering out a subset of variables with non-zero coefficients. In the parameter refitting phase, the selected variables were refitted in an unpenalized logistic regression model to eliminate estimation bias caused by LASSO shrinkage (Meinshausen, 2007).

**Figure 1:**
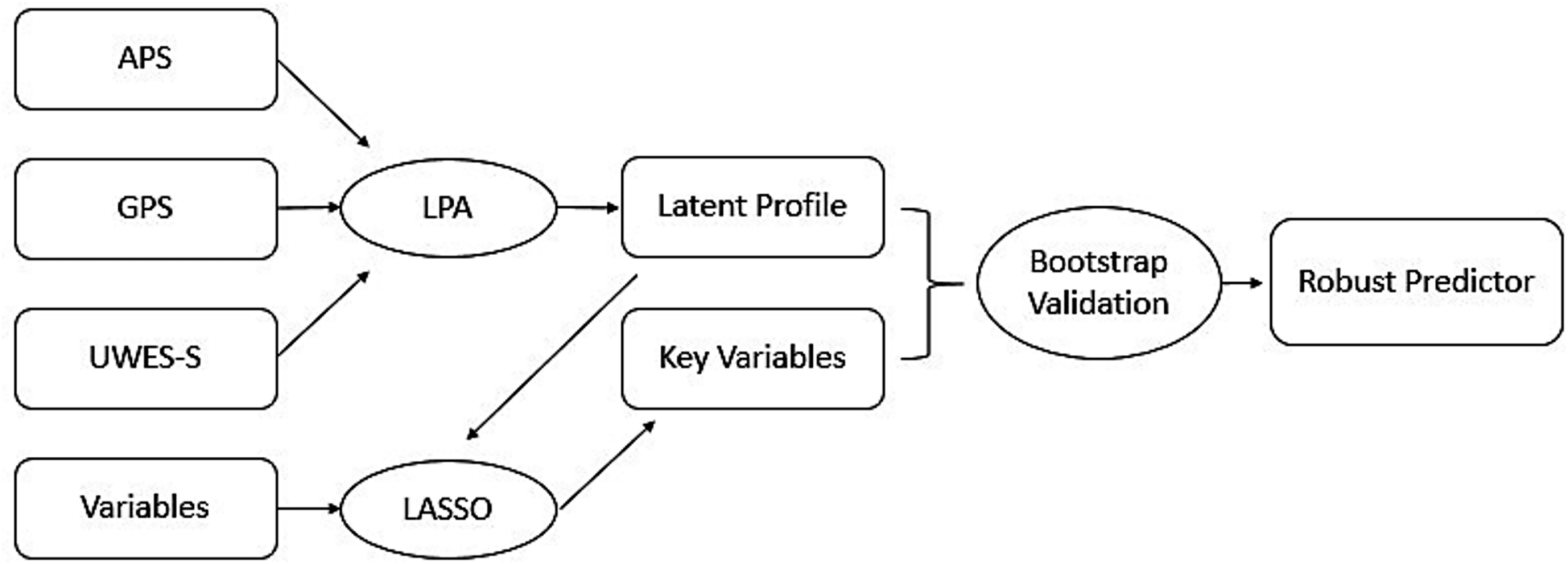
Primary data analysis flowchart.

Finally, results from 1,000 iterations are aggregated. To ensure the robustness of predictive factors, this study employs variable selection based on the “majority voting rule” in ensemble feature selection (Pes, 2020), variable importance is measured by selection frequency at a 50% inclusion threshold (Khaire and Dhanalakshmi, 2022). Effect size is reported as the median odds ratio (OR) of the re-estimated coefficients. Statistical significance is assessed based on the bootstrap empirical distribution, reporting 95% confidence intervals and empirical *p*-values. These analyses are primarily implemented using the glmnet and boot packages in R. The primary data analysis pipeline of this study is shown in Figure 1.

## Results

### Sample characteristics and measurement assessment

In this study, we collected data from 517 college students. Regarding basic demographic characteristics, males accounted for 43.7% (226), while females constituted 56.3% (291). The ethnic composition was predominantly Han Chinese, representing 98.6% (510). Regarding political belief, 55.3% (286) were youth league member, while 37.3% (193) were the general public. In terms of academic background and experience, the sample primarily consisted of second-year (44.7%) and third-year (44.5%) undergraduates.

The majority had a background in science-focused high school tracks (59.4%). The vast majority of students (80.9%) took the Gaokao only once. Over 60% (61.9%) had served as student leaders. Most students (80.1%) did not change their majors during university. Regarding personal behavior and family background, 77.6% of students participated in volunteer service. The median number of late bedtime frequency was 6, while the median number of purposeful exercise frequency was 3. Regarding scale scores, the median scores (interquartile range) were as follows: FAS-III 6 (Barbayannis et al., 2022; Bandura, 1977), GSES 29 (Nylund-Gibson et al., 2023; Aquino et al., 2025), PSSS 60 (49, 71), APS 30 (Nylund-Gibson et al., 2023; Lay, 1986) GPS 30 (Nie et al., 2024; Li and Huang, 2010), and UWES-S 68 (63, 77). Detailed data are presented in Table 2.

**Table 2:** General characteristics.

Variables		Total [*n* = 517;n(%)]
Gender	Male	226(43.7)
	Female	291(56.3)
Ethnic	Han	510(98.6)
	Ethnic minorities	7(1.4)
Political belief	General Public	193(37.3)
	Youth league member	286(55.3)
	Student party	38(7.4)
Academic year	First-year	37(7.4)
	Second-year	231(44.7)
	Third-year	230(44.5)
	Senior	19(3.7)
High school track	Liberal arts track	191(36.9)
	Science track	307(59.4)
	No division	19(3.7)
Number of Gaokao attempts	1	418(80.9)
	2	94(18.2)
	3 or more	5(1.0)
Scholarships(past year)	Yes	88(17.0)
	No	429(83.0)
English proficiency	CET-4	189(36.6)
	CET-6	45(8.7)
	Neither	283(54.7)
Student leadership experience	Yes	320(61.9)
	No	197(38.1)
Current student leadership position	Yes	195(37.7)
	No	322(62.3)
Major change	Yes	103(19.9)
	No	414(80.1)
Military service	Yes	5(1.0)
	No	512(99.0)
Volunteer service	Yes	401(77.6)
	No	116(22.4)
Father’s educational attainment	Primary	61(11.8)
	Middle school	243(47.0)
	High school or above	213(41.2)
Mother’s educational attainment	Primary	98(19.0)
	Middle school	230(44.5)
	High school or above	189(36.6)
Late bedtime frequency	Median(P25, P75):6(3.7)	
Purposeful exercise frequency	Median(P25, P75):3(1.5)	
FAS-III	Median(P25, P75):6(4.8)	
GSES	Median(P25, P75):29(26.30)	
PSSS	Median(P25, P75):60(49.71)	
APS	Median(P25, P75):30(26.32)	
GPS	Median(P25, P75):30(24.35)	
UWES-S	Median(P25, P75):68(63.77)	

Gaokao: national college entrance examination, is a standardized high-stakes exam that serves as the primary criterion for admission into virtually all Chinese universities. Late bedtime frequency: the number of days per week going to bed after 11:00 p.m. Purposeful exercise frequency: the number of days per week engaging in purposeful exercise lasting 10 mins or more.

### Latent profile analysis of academic performance

Before identifying the latent profiles, we checked the psychometric properties of all measurement scales. The results of two different statistical analyses, CFA and Harman’s single-factor test, showed that the model fit well, was reliable, and did not have severe common method bias (see details in Supplementary Tables S1–S3).

#### Determination of the number of latent profiles

Our LPA, ranging from 2 to 5 classes, generated models whose fit indices are detailed in the Table 3. Across all models, LMR and BLRT were statistically significant. As the number of categories increased, AIC, BIC, and aBIC values gradually decreased. Although models 2, 4, and 5 maintained high entropy (>0.8), considering the proportion of categories, we selected the 2-class model from the perspective of model interpretability.

**Table 3:** LPA fitting indices.

Class	AIC	BIC	aBIC	Entropy	LMR(p)	BLRT(p)	Sample proportion(%) per class
2	4296.079	4436.033	4306.817	0.887	<0.001	0.01	9.3/90.7
3	4293.494	4348.559	4308.528	0.708	>0.05	0.04	15.4/69.8/14.8
4	4174.515	4268.979	4193.844	0.946	<0.001	0.01	8.8/4.3/86.7/0.2
5	4141.770	4235.227	4165.395	0.0.842	<0.001	0.01	33.3/6.4/2.2/44.2/13.9

#### Latent profile naming and characteristics

**Figure 2:**
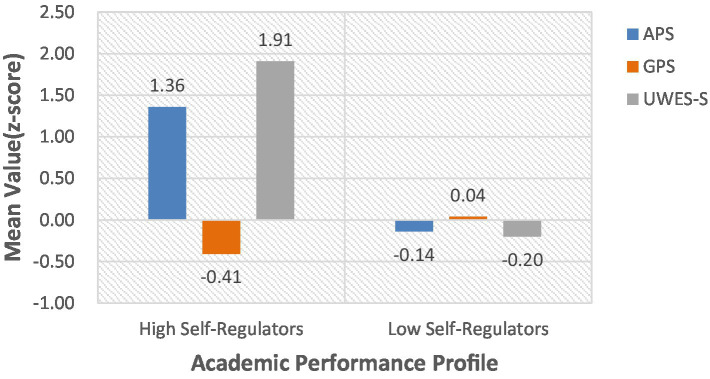
Mean standardized scores across three scales for the two profiles.

Based on standardized scores across the three scales (Figure 2), category 1 (*n* = 42, 8.1%) was defined as the High Self-Regulators, while category 2 (*n* = 475, 91.9%) was defined as the Low Self- Regulators. Students in the first category exhibit higher APS and UWES-S scores but lower GPS scores, with median APS and UWES-S scores of 37 and 106, respectively, and a median GPS score of 24. The second group of students scored average on APS and UWES-S but higher on GPS, with median APS and UWES-S scores of 29 and 68, respectively, and a median GPS score of 30.

#### Comparison of characteristics across latent profiles

Univariate analysis results indicate (Table 4) that different latent categories of academic performance exhibit significant differences in mother’s educational attainment, purposeful exercise frequency, FAS-III, GSES, and PSSS. For variables such as ethnicity, political belief, academic year, high school track, number of Gaokao attempts, English proficiency, major change, military service, volunteer service, father’s educational attainment, and mother’s educational attainment. Due to extremely small sample sizes (<10 individuals) in specific categories and concerns that data sparsity may compromise the robustness of multivariate models, these variables are included only for descriptive analysis and excluded from subsequent LASSO and regression analyses. It is noteworthy that among these excluded variables, significant differences exist across academic performance categories for volunteer service and mother’s educational attainment. Results for the remaining variables indicate no significant distribution differences across categories.

**Table 4:** Univariate analysis of latent categories of academic performance.

Variable		Low self-regulators [*n* = 475;n(%)]	High self-regulators [*n* = 42;n(%)]	Statistics	*p* value
Gender	Male	207(43.6)	19(45.2)	0.00^a^	0.964
	Female	268(56.4)	23(54.8)		
Ethnic	Han	468(98.5)	42(100)	F	1
	Ethnic minorities	7(1.5)	0(0)		
Political belief	General Public	34(7.2)	13(31)	F	0.605
	Youth league member	261(54.9)	25(59.5)		
	Student party	180(37.9)	4(9.5)		
Academic year	First-year	36(7.6)	1(2.4)	F	0.575
	Second-year	213(44.8)	18(42.9)		
	Third-year	209(440)	21(50)		
	Senior	17(3.6)	2(4.8)		
High school track	Liberal arts track	176(37.1)	15(35.7)	F	0.955
	Science track	281(59.2)	26(61.9)		
	No division	18(3.80)	1(2.4)		
Number of Gaokao attempts	1	384(80.8)	34(81)	F	0.894
	2	86(18.1)	8(19)		
	3 or more	5(1.1)	0(0)		
Scholarships(past year)	Yes	77(16.2)	11(26.2)	2.06^a^	0.151
	No	398(83.8)	31(73.8)		
English proficiency	CET-4	18(42.9)	171(36)	F	0.381
	CET-6	5(11.9)	40(8.4)		
	Neither	19(45.2)	264(55.6)		
Student leadership experience	Yes	290(61.1)	30(71.4)	1.35^a^	0.245
	No	185(38.9)	12(28.6)		
Current student leadership position	Yes	175(36.8)	20(47.6)	1.48^a^	0.224
	No	300(63.2)	22(52.4)		
Major change	Yes	97(20.4)	6(14.3)	0.57^a^	0.452
	No	378(79.6)	36(85.7)		
Military service	Yes	4(0.8)	1(2.4)	F	0.346
	No	471(99.2)	41(97.6)		
Volunteer service	Yes	362(76.2)	39(92.9)	5.23^a^	0.022
	No	113(23.8)	3(7.1)		
Father’s educational attainment	Primary	58(12.2)	3(7.1)	F	0.187
	Middle school	227(47.8)	16(38.1)		
	High school or above	190(40)	23(54.8)		
Mother’s educational attainment	Primary	95(20)	3(7.1)	6.66^a^	0.036
	Middle school	213(44.8)	17(40.5)		
	High school or above	167(35.20)	22(52.4)		
Late bedtime frequency	Median(P25, P75)	6(4–7)	5(3–7)	–1.19^b^	0.221
Purposeful exercise frequency		2 (1, 5)	4 (2,7)	3.43^b^	<0.001
FAS-III		6(4, 8)	7.5(5.2,10)	2.92^b^	0.003
GSES		29 (25.5, 30)	33.5 (30,38)	7.13^b^	<0.001
PSSS		60 (48, 70)	71(65,72)	4.97^b^	<0.001

a χ^2^ and Chi-square tests.

b Z value and Mann–Whitney U tests, F, fisher’s exact tests.

#### Predictors of latent profile membership

**Figure 3:**
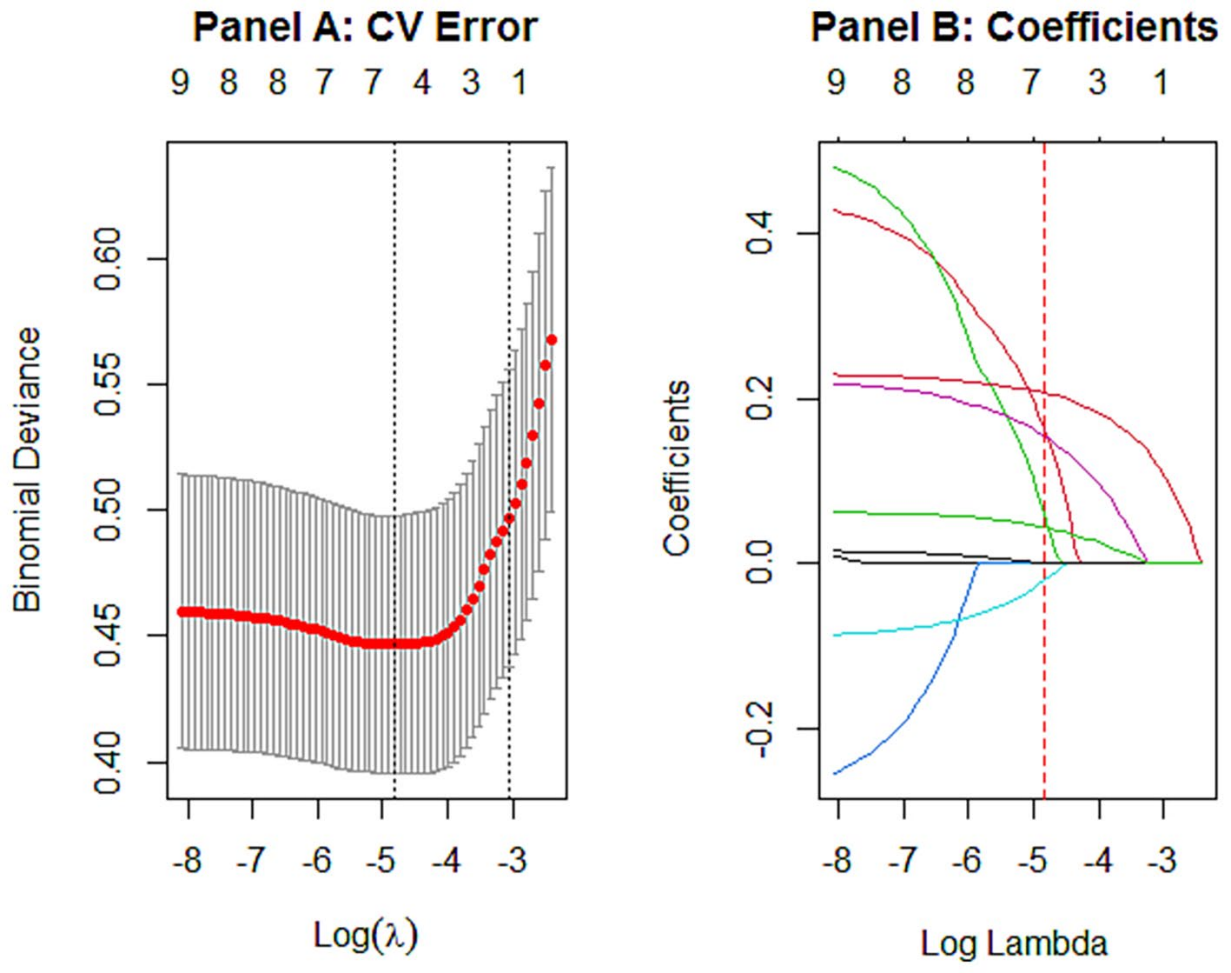
The dynamic process of variable selection via LASSO regression. Panel **A**, cross-validation error (binomial deviance) plotted against the penalty coefficient log(*λ*). Panel **B**, coefficient profiles of the 9 candidate variables, the coefficient paths show the shrinkage of variables toward zero as the penalty increases.

Variable selection via bootstrap LASSO. To avoid multicollinearity among variables and enhance model stability, this study employed bootstrap relaxed LASSO regression for feature selection. Candidate variables included gender, scholarships (past year), student leadership experience, current student leadership position, purposeful exercise frequency, late bedtime frequency, FAS-III, PSSS, and GSES, excluding sparse variables. Figure 3 illustrates the parameter tuning and dynamic dimensionality reduction process of the LASSO model. Panel A shows the fluctuation of cross-validation error as the penalty coefficient varies. Panel B displays the shrinkage paths of variable coefficients. As depicted, increasing penalty strength compresses coefficients of less influential variables to zero, achieving automatic feature reduction.

**Figure 4:**
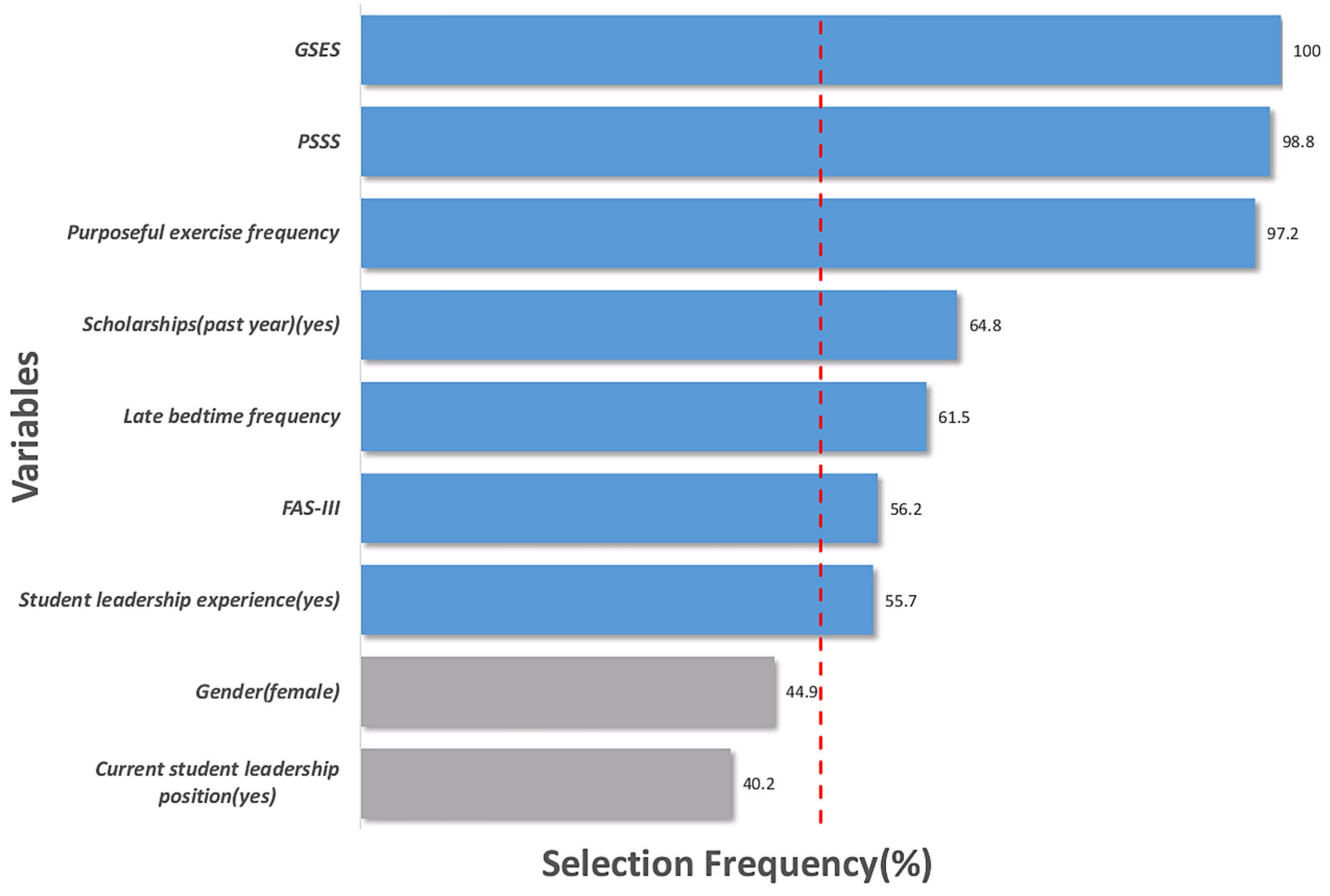
Robustness assessment of predictors based on selection frequency. Note: The horizontal bars represent the percentage of times each variable was selected across 1,000 bootstrap LASSO replicates, variables with higher frequency indicate greater stability.

Based on the above screening, Figure 4 displays the frequency with which each variable was selected during 1,000 bootstrap resamples, reflecting the core predictive power and robustness of each predictor for the latent categories of academic performance. Among these, the selection frequencies for GSES, PSSS, purposeful exercise frequency, scholarships (past year), late bedtime frequency, FAS-III, and student leadership experience were all below 50%.

### Logistic regression results

Based on 1,000 bootstrap resamples, we assessed the stability of predictor variables. Results indicate that GSES, PSSS, and purposeful exercise frequency demonstrate exceptionally high stability in the penalized model, each selected by the model over 90% of the time. Considering statistical significance, these three variables are identified as core predictors influencing the latent categories of academic performance. Specifically, the model using the “High Self-Regulators” as a reference group, each 1-point increase in GSES scores significantly reduced the odds of belonging to “Low Self-Regulators” by 21%. Each additional day of purposeful exercise frequency decreased the odds of belonging to “Low Self-Regulators” by 19%. Meanwhile, each 1-point increase in PSSS scores correspondingly lowered the odds of becoming “Low Self-Regulators” by 7%. Detailed data are presented in Table 5.

**Table 5:** Robustness assessment and effect estimates of predictors based on Bootstrap penalized regression.

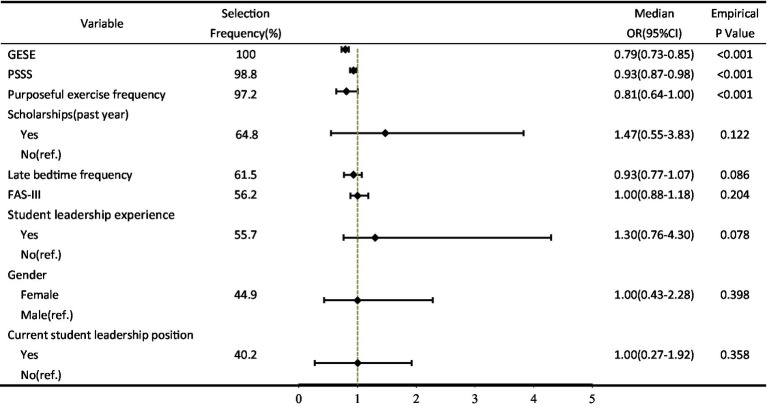

## Discussion

### Summary of main findings

This study adopts a learner-centered perspective to explore the underlying heterogeneity in college students’ academic performance. We identified two latent categories of academic performance among college students through LPA analysis, naming them the “Low Self-Regulators” and the “High Self-Regulators.” This distinction reveals significant differences in learning regulation and achievement performance among college students, supporting the notion of group heterogeneity in academic behavior, that is, different students adopt distinct psychological and behavioral pathways in pursuing learning goals (Zhao and Ling, 2022; Jeong and Feldon, 2023). Building upon this foundation, the study further examined demographic and psychological factors potentially influencing group affiliation by employing the more robust variable selection technique known as Bootstrap Relaxed LASSO. Analysis indicates that GESE, PSSS, and purposeful exercise frequency significantly predict students’ latent category affiliation. These findings not only deepen our understanding of group heterogeneity in academic performance among students but also lay the groundwork for subsequent targeted interventions to enhance their academic achievement and mental health.

### Latent category characteristics of college students’ academic performance

The “Low Self-Regulators” accounted for 91.9% of participants. They scored average on the APS and UWES-S scales but had higher GPS scores. Higher GPS scores indicate severe abnormalities in behavioral control among such students. This finding aligns with prior research, where procrastination is regarded as a primary failure in self-regulation and is associated with deficits in volitional control and inadequate time management planning (Lourenço and Paiva, 2024). This finding effectively explains the situation of Low Self-Regulators. Despite maintaining average levels of academic engagement, their lack of regulatory capacity likely constitutes a cognitive and behavioral bottleneck. This regulatory failure can undermine consistent academic functioning (Ragusa et al., 2023). Consequently, these students remain in a state of average APS. Due to the disruptive nature of procrastination, they are unable to fully translate their efforts into effective academic performance.

The “High Self-Regulators” accounted for 8.1% of participants. They scored higher on the APS and UWES-S scales and lower on the GPS scale. From the perspective of SRL theory, this profile represents the comprehensive integration of the regulatory dimension. This alignment is consistent with extensive meta-analytic evidence indicating that motivation and self-regulation strategies function as mutually reinforcing components of academic success (Theobald, 2021). More specifically, their strong motivation and higher learning engagement appear to effectively support volitional control, lower procrastination, thereby promoting the efficient execution of adaptive strategies, yielding higher APS scores. These dimensions do not operate in isolation but function synergistically to exert their effects. This consistency characteristic reflects a virtuous cycle, psychological investment can enhance volitional regulation, ultimately facilitating effective academic performance.

### Influencing factors of latent categories in college students’ academic performance and educational implications

On the psychological front, “Low Self-Regulators” and “High Self-Regulators” exhibit significant differences in self-efficacy. Specifically, while the “Low Self-Regulators” group demonstrates average academic performance and learning engagement, their self-efficacy levels are markedly lower and accompanied by higher levels of procrastination. This finding aligns with previous research conclusions. Lower self-efficacy implies individuals lack confidence and a sense of control in learning, making them more prone to maladaptive behaviors such as avoidance and procrastination (Rad et al., 2025). Consequently, this group exhibits average academic performance coupled with higher levels of procrastination, indicating their reliance on high-cost strategies (Sirois, 2023). Meta-analytic research suggests this pattern is likely sustained by introjected regulation, a motivation driven by internal pressure, guilt, and anxiety that maintains academic performance but imposes significant psychological strain (Howard et al., 2021). In contrast, the higher self-efficacy of “High Self-Regulators” students foster a virtuous cycle of “high engagement and high achievement” (Talsma et al., 2018), enabling them to sustain effective learning efficiency and academic performance through intrinsic motivation. Therefore, educators should be attentive to students who appear average academically, as their academic performance may be indicative of significant psychological stress. By enhancing their sense of self-efficacy, it is possible to transform their learning approach from a high-maintenance, anxiety-driven struggle into an efficient, interest-based pursuit, thereby ensuring long-term academic resilience.

Additionally, PSSS was identified as a significant predictor influencing college students’ latent academic profile membership. Several studies also support the consensus that social support serves as a crucial external resource for college students. Specifically, social support positively predicts academic engagement and self-efficacy while acting as a protective buffer against academic procrastination (Liu, 2024; Chen et al., 2025). Our findings support these conclusions. Analysis shows that students who have more social support are less likely to become Low Self-regulators. This suggests that not having enough social support is a big risk factor for the Low Self-regulators profile, while having strong social support is a key difference for High Self-regulators, helping them to do better and adapt. Therefore, higher education institutions should create collaborative learning environments for students, strengthen peer connections, and enhance teacher-student interactions. This approach provides students with assistance and support, effectively mitigating academic stress, reducing the risk of procrastination stemming from isolation, and promoting better academic performance.

Beyond psychological factors, this study also identified differences in lifestyle variables across distinct latent categories. Students classified as “Low Self-Regulators” engaged in purposeful exercise significantly less frequently than their “High Self-Regulators” counterparts. This finding aligns with extensive prior research demonstrating the cognitive and self-regulatory benefits of physical activity. Specifically, regular exercise not only correlates positively with executive function but also enhances self-regulatory capacity (Zhao et al., 2024). Furthermore, physical exercise has been shown to significantly improve emotional regulation skills (Liu et al., 2022). These exercise-induced enhancements in psychological and cognitive functioning con tribute to strengthened self-control, ultimately influencing academic performance (Cai et al., 2025). It can be inferred that the weaker self-regulation abilities (high procrastination) exhibited by “Low Self-Regulators” in this study may be partially attributable to insufficient physical activity. This also implies that promoting consistent exercise habits among college students could serve as a viable intervention pathway to improve self-regulation and reduce procrastination.

Overall, this study reveals underlying psychological and behavioral differences among college students that influence academic performance. Self-efficacy, perceived social support and purposeful exercise frequency collectively shape distinct learning regulation types. In educational practice, teachers and university counselors can develop differentiated support strategies for different student categories based on this research. For instance, time management training, emotional regulation guidance, and self-efficacy interventions can enhance learning efficiency for students with “Low Self-Regulators.” Drawing on evidence from broader educational settings, interventions centered on mindfulness also offer a promising strategy. While often highlighted in primary and secondary education for reducing school refusal and emotional difficulties (Sorrenti et al., 2025; Sorrenti et al., 2025; Bockmann and Yu, 2023), the core mechanism of mindfulness involves increasing awareness of present experiences, a process that is equally applicable to university students. By fostering such awareness, these interventions can help college students mitigate maladaptive strategies like procrastination and cultivate greater academic resilience. While “High Self-Regulators” may benefit from more challenging tasks and goal-oriented support to sustain their motivation and sense of achievement.

Future longitudinal studies could further examine the academic trajectories of these student categories. Combined with experimental interventions, such research could evaluate the long-term effects of self-regulation training and exercise programs. This would not only deepen our understanding of the mechanisms underlying academic behavior differentiation but also provide an evidence-based foundation for academic guidance and psychological support in higher education.

### Limitations

This study has several limitations that warrant consideration. First, participants were recruited through convenience sampling, and approximately 90% came from a comprehensive university in Henan Province. This may limit the generalizability of the study findings. Second, all scales employed were self-report questionnaires, which may be influenced by social desirability, memory bias, and psychological states, leading to under or overestimation of students’ actual academic behaviors or procrastination levels. Finally, the cross-sectional design precludes establishing causal relationships between academic performance categories and the variables examined.

## Conclusion

This study adopts a person-centered research perspective and employs a multidimensional statistical framework to systematically identify latent categories of college students’ academic performance and the key factors influencing these latent categories. The study identified two distinct student groups: “Low Self-Regulators” and “High Self-Regulators.” Further analysis revealed that general self-efficacy, perceived social support, and deliberate practice frequency are the most critical indicators distinguishing these two groups.

This finding validates the unique value of the person-centered perspective in educational assessment, which transcends linear relationships between single variables to reveal students’ complex psychological and behavioral patterns. Accordingly, higher education institutions should implement differentiated support strategies, providing targeted interventions for specific student groups to effectively enhance their academic performance.

## Data Availability

The original contributions presented in the study are included in the article/Supplementary material, further inquiries can be directed to the corresponding author.
