# Supplementary material for: Exploring heterogeneous profiles of academic performance among college students: a latent profile and LASSO regression analysis
Source: Front Psychol. 2026 May 13;17:1732350. doi: 10.3389/fpsyg.2026.1732350 (PMC13212273; doi:10.3389/fpsyg.2026.1732350)
Supplement: Supplementary file 1 [file Data_Sheet_1.PDF]

Supplementary Information(SI)

Exploring Heterogeneous Profiles of Academic Performance Among College Students: A Latent Profile and LASSO Regression Analysis

Supplementary tables:

Table S1 Mode fit indices for the measurement model and common method bias test.

Model	χ^2	*df*	χ^2 /*df*	CFI	TLI	RMSEA	SRMR
Hypothesized model (5-factor)	1131.22	549.00	2.06	0.93	0.93	0.05	0.05
Harman's single-factor model	5430.16	559.00	9.71	0.42	0.39	0.16	0.18

Note. N =517. Estimation method: Robust Maximum Likelihood (MLR). CFI = Comparative Fit Index, TLI = Tucker-Lewis Index, RMSEA = Root Mean Square Error of Approximation, SRMR = Standardized Root Mean Square Residual. The Hypothesized Model includes five latent factors: General Self-Efficacy Scale(GSES), Perceived Social Support Scale(PSSS), Academic Performance Scale(APS), General Procrastination Scale(GPS), Utrecht Work Engagement Scale-Student (UWES-S).

Table S2 Psychometric properties of the measurement scales.

Latent Construct	Items	Sta. factor loading	Cronbach's α	CR	AVE
GESE	Item1	0.65	0.93	0.93	0.57
	Item2	0.65			
	Item3	0.73			
	Item4	0.83			
	Item5	0.81			
	Item6	0.72			
	Item7	0.81			
	Item8	0.78			
	Item9	0.75			
	Item10	0.78			
PSSS	Family	0.85	0.92	0.92	0.80
	Friend	0.91			
	Other	0.93			
APS	Item1	0.76	0.90	0.90	0.54
	Item2	0.79			
	Item3	0.56			

	Item4	0.83			
	Item5	0.71			
	Item6	0.69			
	Item7	0.78			
	Item8	0.78			
GPS	Item1	0.67	0.92	0.91	0.52
	Item2	0.75			
	Item5	0.64			
	Item7	0.84			
	Item9	0.83			
	Item10	0.75			
	Item11	0.70			
	Item12	0.77			
	Item16	0.60			
	Item17	0.67			
	Item19	0.69			
UWES-S	Vigor	0.89	0.94	0.94	0.85
	Dedication	0.94			
	Absorption	0.93			

Note: Parcels: PSSS and UWES-S are item parcels created by averaging items within their theoretical sub-dimensions. Refinement: GPS items with standardized loadings < 0.50 were excluded from the analysis to optimize construct validity. Correlated Errors: The measurement model allows for correlated residuals between item16 and item16 in the GPS due to semantic overlap. CR = Composite Reliability, AVE = Average Variance Extracted. All factor loadings are significant at p < .001.

Table S3 Descriptive statistics and spearman correlations among study variables.

Variable	Mdn	IQR	GSES	PSSS	APS	GPS	UWES-S
GSES	2.90	0.40	-				
PSSS	5.00	1.83	0.32***	-			
APS	3.75	0.75	0.42***	0.43***	-		
GPS	2.73	1.00	0.03	-0.13**	-0.12**	-	
UWES-S	4.00	0.84	0.39***	0.33***	0.47***	-0.17***	-

Note: Mdn = Median; IQR = Interquartile Range. Values below the diagonal are Spearman's rank correlation coefficients. ***p < 0.001, ** 0.001 <p < 0.01, * p <0 .05. Non-significant correlation was observed between GSES and GPS (r = 0.03, p = 0.489), indicating potential population heterogeneity that supports the use of Latent Profile Analysis.
